# Clinical Application of Blood Lactate, C‐Reactive Protein, and the Age‐Adjusted Charlson Comorbidity Index in Predictive Assessment of Malignant Risk of MRSA Pneumonia

**DOI:** 10.1155/mi/8850275

**Published:** 2026-07-08

**Authors:** Heqing Huang, Hong Lu, Yiling Chen, Qiongfang Zhu, Yalu Ren, Zutao Chen, Jie Xu

**Affiliations:** ^1^ Center of Clinical Laboratory, The First Affiliated Hospital of Soochow University, Suzhou, China, sdfyy.cn; ^2^ Department of Infectious Diseases, The First Affiliated Hospital of Soochow University, Suzhou, China, sdfyy.cn; ^3^ MOE Key Laboratory of Geriatric Diseases and Immunology, Suzhou Key Laboratory of Pathogen Bioscience and Anti-Infective Medicine, Soochow University, Suzhou, China, scu.edu.tw

**Keywords:** aCCI score, blood lactate, CRP, early warning model, MRSA, severe pneumonia

## Abstract

**Background:**

The objective of this study is to develop an early warning model that combines the age‐adjusted Charlson comorbidity index (aCCI) with serum lactate (Lac) and C‐reactive protein (CRP) levels to predict the progression of early methicillin‐resistant *Staphylococcus aureus* (MRSA) pneumonia into severe pneumonia (SP).

**Methods:**

This study was a retrospective case–control analysis. Sixty‐four inpatients diagnosed with MRSA pneumonia at the First Affiliated Hospital of Soochow University from November 2023 to August 2024 were selected as study subjects. They were divided into two groups: the SP group (SP group) and the non‐SP (nSP) group, based on established diagnostic criteria for SP. Demographic, clinical, and laboratory data of the patients were collected and statistically analyzed.

**Results:**

Twenty‐four patients (37.5%) developed SP, and the aCCI score was significantly higher in the SP group compared to the nSP group, with a statistically significant difference between the two groups (*p*  < 0.05). Hospital‐associated pneumonia caused by hospital‐acquired MRSA (HA‐MRSA) was more prevalent in the nSP group (*p*  < 0.05). However, the differences between the SP and nSP groups were not statistically significant for laboratory indicators such as white blood cell (WBC) count, lymphocyte (LY) count, neutrophil (NE) count, and platelet (PLT) count (*p*  > 0.05). The levels of CRP and serum Lac in the SP group were significantly higher than those in the nSP group, with statistically significant differences (*p*  < 0.05). Multifactorial logistic regression analysis indicated that HA‐MRSA was an independent protective factor against MRSA‐associated SP, while the aCCI score, CRP, and serum Lac levels were identified as risk factors for the progression of MRSA to SP. The combination of these three factors demonstrated good predictive efficacy, with an area under the curve (AUC) of 0.913 (*p*  < 0.001, 95% CI: 0.841, 0.985).

**Conclusion:**

MRSA strains associated with SP demonstrate a lack of specificity in their clinical manifestations. HA‐MRSA is less likely to lead to SP compared to community‐acquired MRSA (CA‐MRSA). An aCCI score ≥7, in conjunction with elevated levels of early CRP and serum Lac, can serve as effective early indicators that MRSA pneumonia may progress to a severe form.

## 1. Introduction

Methicillin‐resistant *Staphylococcus aureus* (MRSA) is one of the most successful modern pathogens that is resistant to several antibiotics. Following invasion, it can lead to a variety of infections, including osteomyelitis, endocarditis, skin and soft tissue infections, and pneumonia. Although the prevalence of pneumonia caused by MRSA is relatively low [1], MRSA pneumonia tends to result in more severe clinical outcomes compared to infections caused by *Streptococcus pneumoniae*. Furthermore, MRSA remains the most significant gram‐positive coccus associated with hospital‐acquired infections. Severe pneumonia (SP) arises from the progression of lung tissue inflammation to an advanced stage of the disease, leading to deterioration and exacerbation, which can result in organ dysfunction or even be life‐threatening.

In recent years, studies have shown [2, 3] that the probability of MRSA causing SP is increasing annually. Meanwhile, Carey et al. [1] concluded that the empirical use of anti‐MRSA antimicrobial agents does not significantly reduce the mortality rate associated with MRSA infections. Therefore, it is essential to develop an early warning model to assess the risk of deterioration in MRSA pneumonia during its initial stages. Although the classical pneumonia severity index (PSI) score and CURB‐65 score are effective in predicting 30‐day mortality, they are less reliable for the early assessment of the potential progression to SP [4].

C‐reactive protein (CRP) is a well‐established inflammatory marker that can increase rapidly during the acute phase of inflammation. Lactate (Lac), an intermediate product of glucose metabolism, can serve as an indicator of infection severity when hyperlactatemia is present [5]. The age‐adjusted Charlson comorbidity index (aCCI) was utilized to evaluate the impact of patients’ comorbidities and underlying conditions on their survival. This index is particularly relevant for middle‐aged and elderly hospitalized patients, who often present with a range of comorbid diseases of varying severity. Originally applied in the prognostic assessment of oncology patients, the Charlson [6] score is now increasingly used in the context of infections.

In order to integrate common inflammatory indicators, microcirculatory status, and patients’ underlying comorbidities to assess the severity of infections, we have developed an early warning model for the rapid identification of the risk of early deterioration in MRSA pneumonia. This model enables timely clinical intervention and treatment to prevent adverse consequences.

## 2. Materials and Methods

### 2.1. Study Design

Sixty‐four patients with MRSA‐associated pneumonia were admitted to the First Affiliated Hospital of Soochow University from November 2023 to August 2024. These patients were diagnosed with pneumonia in accordance with the guidelines established by the Infectious Diseases Society of America (IDSA) and the American Thoracic Society (ATS) [7, 8]. Among them, 24 patients were classified as having SP (the SP group), while 40 patients were categorized as having the non‐SP (nSP) group. Inclusion criteria were as follows: (1) age greater than 18 years; (2) fulfillment of the diagnostic criteria for MRSA pneumonia, confirmed through clinical evaluation and differential diagnosis; and (3) meeting the diagnostic criteria for SP in the SP group. Exclusion criteria included (1) substandard quality of retained sputum culture samples; (2) patients with severe immune system disorders or those undergoing long‐term glucocorticoid therapy; (3) patients whose initial sputum cultures revealed pathogenic bacteria in conjunction with other bacterial infections; (4) patients admitted with non‐infectious shock; (5) patients with acute, extensive trauma; and (6) patients with missing data exceeding 20%. Community‐acquired pneumonia (CAP) is defined as an infectious inflammation of the lung parenchyma (including the alveolar walls, corresponding to the pulmonary interstitium in a broad sense) that is acquired outside the hospital. This includes pneumonia caused by pathogens with a defined incubation period that manifests during the incubation period after hospital admission. Hospital‐acquired pneumonia (HAP) refers to pneumonia that develops 48 h or more after hospital admission in patients who have not undergone invasive mechanical ventilation during their stay and were not in the incubation period of a pathogen infection upon admission. Clinical diagnostic criteria: chest X‐ray or CT reveals new or progressive infiltrates, consolidation, or ground‐glass opacities, along with at least two of the following three clinical signs or symptoms: fever with a body temperature greater than 38°C, purulent airway secretions, and peripheral blood leukocyte count >10 × 10^9^/L or <4 × 10^9^/L, along with an etiological diagnosis. Diagnostic criteria for SP: At least one of the following major criteria or three or more minor criteria must be met. Primary criteria: (1) tracheal intubation requiring mechanical ventilation and (2) the need for vasoactive drugs following aggressive fluid resuscitation for infectious shock. Secondary criteria: (1) respiratory rate > 30 breaths/min; (2) PaO_2_/FiO_2_ < 250 mm Hg; (3) White blood cell (WBC) count < 4 × 10^9^/L; (4) platelet (PLT) count < 100 × 10^9^/L; (5) central body temperature < 36°C; (6) pulmonary lobar infiltrates; (7) impaired consciousness and/or disorientation; (8) blood urea nitrogen ≥ 7 mmol/L; (9) and hypotension requiring aggressive fluid resuscitation.

### 2.2. Diagnostic Criteria for MRSA Pneumonia

On the basis of a clinical diagnosis, identification of a causative pathogen is confirmed if one of the following criteria is met, with results consistent with clinical manifestations: isolation of MRSA from qualified lower respiratory tract specimens, protected specimen brush (PSB) obtained via bronchoscopy, and bronchoalveolar lavage fluid (BALF). Sputum smears screened by gram staining show fewer than 10 squamous epithelial cells per low‐power field and more than 25 multinucleated cells per low‐power field under microscopy. Alternatively, sputum samples meet the criterion of a squamous epithelial cell‐to‐multinucleated cell ratio of ≤1 : 2.5 per low‐power field, from which the same pathogen is isolated in two consecutive tests. Quantitative bacterial culture of sputum yields a pathogen count of ≥10^6^ CFU/mL. Pathogenic bacteria isolated from BALF obtained via fiberoptic bronchoscopy must be ≥10^4^ CFU/mL. Alternatively, pathogenic bacteria must be isolated from lower respiratory tract secretions collected using a contamination‐proof specimen brush or contamination‐proof bronchoalveolar lavage; however, for patients with pre‐existing chronic obstructive pulmonary disease (including bronchiectasis), the bacterial count threshold is lowered to ≥10^3^ CFU/mL. All the clinical samples were inoculated on blood agar plate (BAP) and chocolate agar (CHOC) and incubated at 35°C for 48–72 h. Species‐level typing was performed using VITEK Mass Spectrometry.

### 2.3. Data Collection

Clinical data were collected from 64 patients, including age, gender, hypertension, diabetes, influenza virus infection, chronic respiratory disease, and the aCCI score. Clinical manifestations recorded included fever, cough, sputum production, tracheal intubation/incision, and the type of pneumonia (CAP) or hospital‐acquired/ventilator‐associated pneumonia (HAP/VAP). Laboratory data included WBC count (3.5–9.5 × 10^9^/L), lymphocyte (LY) count (1.10–3.20 × 10^9^/L), neutrophil (NE) count (1.80–6.30 × 10^9^/L), CRP (0–4 mg/L), and serum Lac (0.5–1.6 mmol/L). Attention should be paid to ensuring that the collection time is close to the time of the first etiological specimen obtained after admission.

### 2.4. Statistical Analysis

SPSS Version 29.0 statistical software was utilized for data analysis. Normally distributed measurements were expressed as x±s, and a *t*‐test was employed for comparisons between the two groups. Skewed measurements were reported as M(Q1, Q3), with the Mann–Whitney *U* test used for comparisons between the two groups. Count data were analyzed using the chi‐square test or Fisher’s exact test for comparisons between two groups. The predictive value was evaluated through multifactorial logistic regression analysis, and receiver operating characteristic (ROC) curves were plotted using GraphPad Prism to assess subjects’ work characteristics, with a *p*‐value of <0.05 considered statistically significant.

## 3. Results

### 3.1. Comparison of Baseline Data, Clinical Characteristics, and Laboratory Data Between Patients in the SP and nSP Groups

Among 64 patients with MRSA‐associated pneumonia, 24 patients (37.5%) were in the SP group, while 40 patients (62.5%) were in the nSP group. The differences between the two groups regarding gender, age, and comorbid conditions such as hypertension, diabetes mellitus, influenza A/B virus, and chronic respiratory disease were not statistically significant (*p* > 0.05). The aCCI scores in the SP group were significantly higher than those in the nSP group, with a statistically significant difference (*p* < 0.05). Clinical manifestations were most prevalent in both the SP and nSP groups, including fever, cough, and sputum production, followed by tracheal intubation and pneumonectomy. The incidence of these manifestations was notably higher in the nSP group; however, the difference between the two groups was not statistically significant (*p* > 0.05). In the SP group, there were 12 cases of hospital‐acquired MRSA (HA‐MRSA)‐associated pneumonia (50.0%), while the nSP group had 35 cases (87.5%). The difference in incidence between the two groups was statistically significant (*p* < 0.05). WBC count, NE count, LY count, and PLT count did not show statistically significant differences in intergroup comparisons (*p* > 0.05). Conversely, CRP and serum Lac levels were higher in the SP group than in the nSP group, with the difference being statistically significant (*p* < 0.05) (Tables 1 and 2).

**Table 1:** Comparison of baseline data and clinical features between SP group and nSP group (*n* [%]).

Variables	Total (*n* = 64)	SP group (*n* = 24)	nSP group (*n* = 40)	Statistics	*p*‐Value
Male (%)	48 (75.0)	17 (70.8)	31 (77.5)	*χ* ^2^ = 0.356	0.551
Age (years M [P25, P75])	66.0(54.0,80.8)	77.5(52.3,81.8)	61.5(54.0,75.8)	*z* = −1.498	0.134
Complications					
Hypertension (%)	40 (62.5)	15 (62.5)	25 (62.5)	*χ* ^2^ = 0.000	1.000
Diabetes (%)	14 (21.9)	8 (33.3)	6 (15.0)	*χ* ^2^ = 2.950	0.086
Influenza virus(%)	10 (15.6)	7 (29.2)	3 (7.5)	*χ* ^2^ = 3.824	0.051
Chronic respiratory diseases	9 (14.1)	5 (20.8)	4 (10.0)	*χ* ^2^ = 0.698	0.403
aCCI score	5.0 (3.0,7.8)	8.0 (4.0,9.0)	4.0 (3.0,5.0)	*z* = −3.271	0.001
Clinical presentation (*n* [%])					
Fever	44 (68.8)	18 (75.0)	26 (65.0)	*χ* ^2^ = 0.310	0.577
Cough	44 (68.8)	20 (83.3)	24 (60.0)	*χ* ^2^ = 3.801	0.051
Endotracheal intubation	42 (65.6)	14 (58.3)	28 (70.0)	*χ* ^2^ = 0.905	0.341
HA‐MRSA	47 (73.4)	12 (50.0)	35 (87.5)	*χ* ^2^ = 10.814	0.001

*Note: p*‐Value represented the comparison between severe group and nonsevere group. *p*‐Values < 0.05, indicated significant difference between severe group and nonsevere group. M (P25, P75): M represents the median, while P25 and P75 denote the 25th and 75th percentiles, respectively. aCCI score: the age‐adjusted Charlson comorbidity index (aCCI) score. HA‐MRSA, hospital‐acquired pneumonia caused by methicillin‐resistant *Staphylococcus aureus*.

**Table 2:** Comparison of laboratory data between SP group and nSP group.

Variables	Total (*n* = 64)	SP group	nSP group	Statistics	*p*‐Value
WBC(×10^9^/L)	9.82(6.75.12.55)	11.85(7.27,13.67)	9.25(6.69,12.37)	*z* = −0.374	0.708
LYC(×10^9^/L)	0.86 ± 0.37	0.92 ± 0.74	0.99 ± 0.61	*t* = 0.407	0.686
NE(×10^9^/L)	9.35 (5.09,12.28)	9.35 (5.91,12.28)	8.54 (4.43,10.83)	*z* = −0.097	0.923
PLT(×10^9^/L)	185.00 (140.00,305.00)	178.00 (140.00,204.00)	185.00 (148.50,309.50)	*z* = −0.111	0.912
CRP(mg/L)	23.06 (10.71,90.70)	97.88 (32.37,128.77)	14.79 (8.15,30.34)	*z* = −3.707	＜0.001
Lac(mmol/L)	0.80(0.80,1.58)	1.60(0.80,2.35)	0.80(0.80,1.10)	*z* = −3.219	0.001

*Note: p*‐Value represented the comparison between severe group and nonsevere group. *p*‐Values < 0.05, indicated significant difference between severe group and nonsevere group. Lac, serum lactate; LYC, lymphocyte count; NE, neutrophil count; PLT, platelet count; WBC, white blood cell count.

Abbreviation: CRP, C‐reactive protein.

### 3.2. Analysis of Factors Affecting SP in Patients Caused by MRSA

Indicators that were significant in the univariate analysis were included in the multifactorial binary logistic regression model. These indicators included pneumonia type (CAP/HAP/VAP), the aCCI score, CRP levels, and serum Lac concentrations. The results indicated that HA‐MRSA served as an independent protective factor against MRSA‐associated SP. Conversely, higher aCCI scores, elevated CRP levels, and increased serum Lac concentrations were identified as risk factors for the progression of MRSA‐associated pneumonia to SP (*p* < 0.05) (Table 3).

**Table 3:** Multivariate logistic regression of MRSA pneumonia in predicting severe pneumonia.

Variables	*B*	S.E.	Wald	*p*‐Value	OR	95% CI
HA‐MRSA	−3.210	1.191	7.260	0.007	0.040	0.004–0.417
aCCI	0.534	0.193	7.705	0.006	1.707	1.170–2.489
Lac	1.347	0.481	7.846	0.005	3.845	1.498–9.865
CRP	0.018	0.007	6.056	0.014	1.019	1.004–1.034

*Note:* HA‐MRSA, hospital‐acquired pneumonia caused by methicillin‐resistant *Staphylococcus aureus*. aCCI, the age‐adjusted Charlson comorbidity Index (aCCI) score. Lac: serum lactate.

Abbreviation: CRP, C‐reactive protein.

### 3.3. Predictive Value of Risk Factors in the Development of MRSA Pneumonia Leading to SP

**Figure 1:**
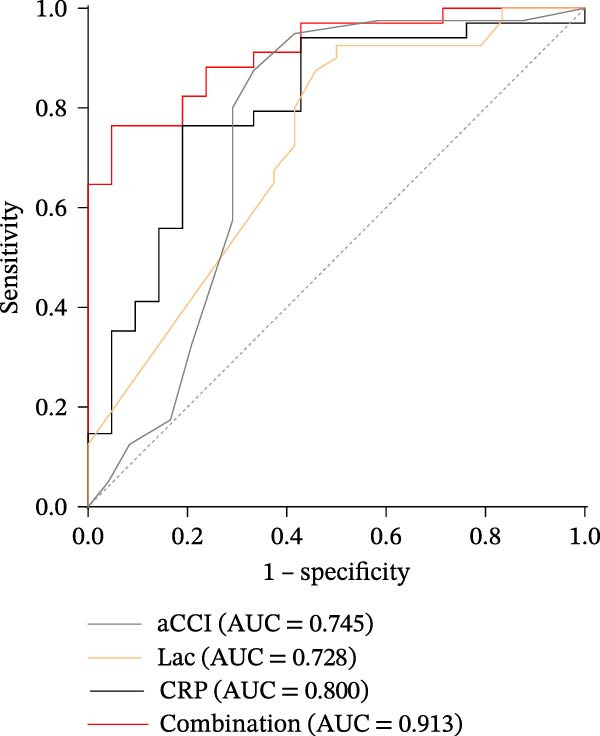
ROC curve of a predictive model for the progression of early methicillin‐resistant *Staphylococcus aureus* (MRSA) pneumonia into severe pneumonia.

Based on the aforementioned risk factors, a ROC curve was constructed to predict the risk of progression in MRSA pneumonia at an early stage. The analysis revealed that the area under the curve (AUC) for the combined predictor—comprising the aCCI score, blood Lac levels, and CRP—was 0.913 (*p* < 0.001, 95% CI: 0.841, 0.985). These results indicate that the combined predictor exhibits significantly superior predictive performance for the deterioration of MRSA pneumonia compared with any individual risk factor (*p*  < 0.05) (Table 4 and Figure 1).

**Table 4:** Predictive value of risk factors for MRSA pneumonia to develop into severe pneumonia.

Variables	AUC	95% CI		*p*‐Value	Truncation value		Sensitivity (%)	Specificity (%)
CRP	0.800	0.675–0.924		<0.001	30.1 mg/L		81.0	73.5
aCCI	0.745	0.590–0.900		<0.001	7 scores		66.7	87.5
Lac	0.728	0.586–0.870		0.002	1.7 mmol/L		50.0	92.5
Combination	0.913	0.841–0.985		<0.001	—		90.5	82.4

*Note:* aCCI, the age‐adjusted Charlson comorbidity index (aCCI) score. Lac: serum lactate.

Abbreviation: CRP, C‐reactive protein.

## 4. Discussion

MRSA is a prevalent and virulent bacterium exhibiting broad‐spectrum resistance in clinical settings. It is currently spreading within communities and hospitals, leading to a high rate of infection. Although the detection rate of MRSA is on a downward trend, some studies indicate that both the rate of MRSA resistance and the incidence of SP caused by MRSA are increasing annually [9, 10]. SP poses a significant threat to human health, and SP caused by MRSA is particularly fatal, with very few clinically available anti‐infective options. Vancomycin and linezolid are the most commonly used treatments in clinical practice. Research has demonstrated [11, 12] that vancomycin remains the standard drug for treating MRSA; however, the gradual rise in the minimum inhibitory concentration (MIC) of vancomycin, along with its nephrotoxicity in recent years, has further complicated its routine use in clinical treatment. Therefore, the early identification of SP caused by MRSA and the implementation of effective treatment strategies are crucial for controlling the disease progression and improving patient outcomes.

The ATS and the IDSA currently recommend the PSI and the CURB‐65 scoring system for assessing the severity of CAP. Although the PSI score is effective in identifying the risk level of pneumonia, it includes too many parameters and is complex to calculate. The CURB‐65 score simplifies the scoring process but reduces sensitivity for predicting 30‐day mortality [13, 14]. In this study, 64 patients were categorized into two groups: the SP group (SP group) and the nSP group, based on whether MRSA pneumonia progressed to SP. A comparison of clinical data revealed statistically significant differences between the SP and nSP groups in the early stages regarding the adjusted Charlson comorbidity index (aCCI) score, HA‐MRSA pneumonia, CRP levels, and serum Lac concentrations. To confirm that these factors were risk indicators influencing the progression of early MRSA pneumonia to SP, they were included in a logistic regression analysis. The results indicated that higher aCCI scores, elevated CRP levels, and increased serum Lac concentrations were associated with a greater likelihood of developing SP. Additionally, HA‐MRSA was identified as an independent protective factor against progression to SP. It has been found [15] that MRSA remains predominantly prevalent in hospitals; however, the frequency of community‐associated infections caused by MRSA is gradually increasing. Historically, community‐acquired MRSA (CA‐MRSA) was primarily linked to skin and soft‐tissue infections, typically resulting in minor illnesses. However, with advancements in the understanding of the epidemiology and molecular biology of MRSA, it is now not uncommon for CA‐MRSA to cause severe infections. CA‐MRSA often produces the cytotoxin Panton–Valentine Leukocidin (PVL), and the morbidity and mortality rate of pneumonia caused by PVL‐positive CA‐MRSA can reach as high as 40% [16]. Furthermore, with the evolving epidemiology of both CA‐MRSA and HA‐MRSA, it is now recognized that HA‐MRSA can also carry the PVL gene. However, in patients with HAP, the severity of the disease and clinical outcomes are not influenced by the presence of the PVL gene. Peyrani et al. [17] concluded that MRSA strains carrying the PVL gene do not express significant levels of the PVL exotoxin in hospitalized patients with HAP or VAP.

The age‐adjusted Charlson score is utilized to evaluate the impact of underlying diseases, aside from the current primary treatment, on patient survival [18]. This assessment aids in accurately determining the severity of the disease. A study [19] found that the aCCI was closely associated with the occurrence and progression of trauma‐related sepsis. CRP, an acute‐phase protein produced by the liver during inflammation, serves as a classical nonspecific biomarker for inflammatory diseases both before and during treatment. CRP levels can increase in response to the severity of the infection. Agrawal and Wu [20] demonstrated that significantly elevated CRP levels in critically ill COVID‐19 patients correlate with disease severity. Furthermore, during bacterial infections, CRP levels are closely related to both the progression and regression of the disease in infected patients. In the present study, CRP levels in the SP group were significantly higher than those in the nSP group, with the difference being statistically significant. Serum Lac is primarily an intermediate product of glucose metabolism in the body, predominantly derived from skeletal muscle, skin, the renal medulla, and erythrocytes. When tissues experience hypoxia, anaerobic metabolism is enhanced, leading to increased Lac levels. Lactic acidosis is a hallmark feature of shock states, and patients with SP often present with hypoxia and hypoperfusion of peripheral circulation, resulting in elevated blood Lac. In recent years, an increasing number of studies have demonstrated that elevated serum Lac levels can serve as indicators of disease severity and mortality assessment. During the immune response, Lac plays a crucial regulatory role in key immune cells, specifically macrophages. Lac can induce epigenetic modifications by binding to macrophage histones, thereby regulating relevant genes and facilitating the transition of macrophages from the proinflammatory M1 type to the anti‐inflammatory M2 type. Under normal circumstances, this mechanism functions as a negative feedback regulation system for macrophages, helping to mitigate the harmful effects of inflammation on the body. However, in cases of severe infection, this process may contribute to immune evasion. Zhang Long et al. [21] discovered that lactic acid can exert an immunosuppressive effect in vivo by mediating the activation of the cGAS. Additionally, Yang et al. [22] found that lactic acid can enhance the accumulation and release of high mobility group box 1 (HMGB1) by promoting the secretion of macrophage exosomes. HMGB1 is a ubiquitous nuclear protein that can be released by activated macrophages to coordinate the inflammatory response, and its levels are positively correlated with the severity of sepsis and mortality. The results indicated that serum Lac and CRP, when combined with the age‐adjusted Charlson score, can effectively predict the risk of worsening MRSA pneumonia at an early stage (AUROC = 0.913). The risk prediction model developed in this study incorporates various dimensions, including the patient’s underlying comorbidities, biomarkers of the inflammatory response, and an assessment of the systemic microcirculatory status. Consequently, it may serve as a valuable screening tool for SP. However, this study also has limitations as the retrospective case–control design includes some missing data and unavoidable biases.

## 5. Conclusion

In summary, aCCI scores, CRP, and blood Lac levels are positively correlated with the severity of MRSA pneumonia. The combined use of these markers (AUC = 0.913, *p*  < 0.001, 95% CI: 0.841–0.985) demonstrates enhanced predictive accuracy for the early progression of MRSA pneumonia to SP. This retrospective, single‐center study is constrained by limitations in sample representativeness and the potential for data bias. Furthermore, the number of SP cases included is insufficient. Future research employing larger sample sizes and multicenter designs is required to improve the generalizability of the findings.

## Funding

The Basic Research Special Project Foundation of Suzhou (Grant SSD2024085) and the Pfizer Foundation (Tracking Number 76080151) supported this work.

## Disclosure

All authors accept the terms and conditions of the editorial for publication.

## Ethics Statement

This study was conducted according to the guidelines of the Declaration of Helsinki and was approved by the Institutional Review Committee of the First Affiliated Hospital of Soochow University (Number 2024056). We confirmed that all data were anonymized and maintained with confidentiality.

## Consent

The required informed consent was waived owing to the retrospective observational nature of the study.

## Conflicts of Interest

The authors declare no conflicts of interest.

## Data Availability

Research data are not shared.
